# Some Causes of Hæmaturia

**Published:** 1909-01-09

**Authors:** 


### SURGERY.

### SOME CAUSES OF HÆMATURIA.

Hæmaturia is a prominent symptom in diseases of the genito-urinary tract, and, moreover, it is often the only symptom of which the patient complains in the early stages. In such cases the first duty of the surgeon is to apply all the means at his disposal for discovering from which portion of that tract the blood is issuing.

First and foremost the history is important and should be ascertained with great care. If the blood is small in amount and comes at the beginning of micturition a urethral cause is suggested. The commonest of such causes, although not so frequent as text-books of surgery might lead one to suppose, is acute gonorrhœa, and this must be excluded. Blood at the end of micturition is likely to be due either to an enlarged prostate or to papilloma of the bladder. The explanation in either case is simple, the hæmorrhage being due to the final contraction of the empty bladder on the diseased part. But if the blood is intimately mixed with the urine and a homogeneous solution is discharged throughout the act, greater difficulty is experienced. If the blood is altered in colour so as to resemble porter, or to be smoky, the condition is probably due to a renal calculus. It is true as a general rule that bright red blood, intimately mixed, is of vesical origin; but the rule must not be regarded as absolute. Tuberculous disease of the kidney which has eroded a largish branch of the renal artery may cause this condition. The hæmorrhage caused by tuberculosis of the kidney is somewhat analogous to hæmoptysis in phthisis. In the early stages in both one gets a capillary oozing; in the case of the kidney the blood so produced is small in amount and is changed in colour as it is in renal calculus. But when, as sometimes happens, a large vessel is eroded, a profuse discharge of bright red blood follows, just as profuse hæmoptysis occurs when an aneurysmal dilatation of some branch of the pulmonary artery gives way.

Most often, however, bright red blood is of vesical origin, and is due either to a papilloma of the bladder or to an ulcer in its wall. An ingenious method has been suggested for making the diagnosis more certain in these cases. A solution of potassium iodide is injected into the bladder, and after a quarter of an hour the saliva is tested with starch solution for the presence of iodine. If there is any abrasion on the surface of the bladder, such as an ulcer, the iodide is rapidly absorbed, but if the bladder-wall is intact this does not happen.

In cases of hæmorrhage due to a papilloma of the bladder, there is one point which is so characteristic as to be almost pathognomonic, and that is the fact that the hæmaturia is intermittent. In a typical case a patient has an attack of transient hæmaturia. Some months later a second attack occurs, and so on, the interval between the attacks becoming gradually shorter and the amount of blood lost on each occasion gradually greater, until finally the patient is rendered blanched and profoundly anæmic. The explanation probably is that as the papilloma increases in size it puts forth a number of slender processes like a sea anemone, and each of these carries a delicate vessel. There comes a time when these processes become so long that their apices are broken off by the contractions of the bladder, and each time that this happens hæmorrhage follows. The increasing frequency of the attacks is due to the growth of the villous tumour and to its tendency to infect the opposite wall of the bladder and become multiple. This may be regarded as an argument in favour of the infective origin of new tissue formation, even though it be innocent; that is if a papilloma of the bladder may be regarded as innocent. Microscopically it may be so, but clinically it can hardly be regarded as such, since it tends to kill the patient in quite a short time if it is not removed.

The passage of elongated clots of blood in the urine is highly suggestive of malignant disease of the kidney. The clots are practically casts of the ureter, though why this should occur in malignant disease more than in other morbid conditions of that organ it is difficult to say.

To make assurance doubly sure the cystoscope should be employed in all cases of hæmaturia, even though the diagnosis appears absolutely certain. Efficiency in using this instrument can only come with practice; and it would profit little to describe the technique in an article of this description. But this much may be said, that if the hæmorrhage is actually going on at the moment, little or nothing can be seen because the bladder is filled with a blood-stained solution which completely obscures the view. In order to conduct a satisfactory cystoscopic examination the bladder must be repeatedly washed out until it contains a clear aqueous solution. The value of the information afforded by the use of this instrument in skilled hands can hardly be over estimated. The condition of the bladder-wall can be thoroughly examined and the presence or absence of a papilloma or an ulcer can be demonstrated. In the absence of any vesical cause, the orifices of the ureters can be examined; and if blood can be seen exuding, it is known not only that it is renal in origin but also from which kidney it is coming. This latter is a point which it is often extremely difficult to decide on the clinical history alone.

Finally, it must be remembered that hæmaturia is occasionally, though rarely, a manifestation of hæmophilia. The writer saw such a case recently. The patient, a man aged thirty, had three attacks of very severe hæmaturia within a month. They came on suddenly and without apparent cause. They were controlled by injection into the bladder of hot water at 115° F., to which was added adrenalin. The case was thought to be one of vesical papilloma. A cystoscopic examination was made, but no abnormality could be found. It was only subsequently that careful questioning elicited that there was a strong family history of hæmophilia, although the patient himself had never previously suffered from unexplained hæmorrhage.

